# Isolated Transaminitis as a Sentinel Sign of Duchenne Muscular Dystrophy in an Infant: A Case Report

**DOI:** 10.7759/cureus.92233

**Published:** 2025-09-13

**Authors:** Ayesha Khalid, Tuba Chaudhry, Ayesha Liaqat, Lauren Tufts

**Affiliations:** 1 Pediatrics, Marshall University Joan C. Edwards School of Medicine, Huntington, USA; 2 Medicine, Surgery, Dermatology, Radiology, Fatima Memorial College of Medicine and Dentistry, Lahore, PAK; 3 Pediatric Emergency Medicine, LSU Health New Orleans School of Medicine, New Orleans, USA

**Keywords:** creatine kinase (ck), duchenne muscular dystrophy, failure to thrive, muscle weakness in infants, neuromuscular disorders, transaminitis, x-linked recessive disorders

## Abstract

Duchenne muscular dystrophy (DMD) is an X-linked recessive muscular dystrophy (MD) that typically presents after ambulation due to progressive proximal muscle weakness. The average age of diagnosis is 4.83 years. However, there are some subtle signs that can help in early diagnosis and delay the progression of the disease. We present a non-classical DMD case in a six-month-old infant with failure to thrive, persistent emesis, transaminitis, and truncal weakness, leading to an early diagnosis of DMD. Initial workup for failure to thrive was unremarkable, other than persistently elevated liver enzymes aspartate aminotransferase (AST) and alanine transaminase (ALT) with normal alkaline phosphatase and bilirubin. Extensive work to rule out gastrointestinal (GI) pathology, lysosomal, and glycogen storage diseases was unremarkable. Significantly elevated creatine kinase (CK: 6,988 U/L), aldolase (>56 U/L), and low alanine (172.4 µmol/L) raised suspicion for MD. Genetic testing confirmed a hemizygous DMD mutation. After nutritional adjustments, the patient gained appropriate weight. He was referred for long-term neuromuscular care. This case underscores the need to recognize atypical DMD presentations, particularly in infants with GI symptoms and unexplained transaminitis. It highlights the importance of CK testing in cases of isolated AST/ALT elevation with a negative GI workup. Early genetic diagnosis is crucial for timely intervention and improved long-term outcomes.

## Introduction

Duchenne muscular dystrophy (DMD) is an X-linked recessive disorder that is more prevalent in males and is among the most common forms of muscular dystrophy. It is typically diagnosed between four and five years of age, often once progressive muscular weakness becomes apparent. However, the literature infrequently reports atypical early presentations, such as failure to thrive or isolated transaminitis, which may be overlooked if the diagnostic focus remains limited to gastrointestinal or hepatic causes [1,2]. We report a non-classical DMD presentation in an infant who presented with failure to thrive, elevated transaminases, and truncal hypotonia, in whom isolated transaminase elevation prompted early discovery of DMD. This case highlights the importance of considering neuromuscular pathologies and obtaining creatine kinase (CK) and genetic testing in infants presented with unexplained elevated liver enzymes [3,4].

## Case presentation

A six-month-old full-term male was admitted for evaluation of persistent emesis and elevated transaminase levels requiring a gastrointestinal (GI) workup. The mother reported that, since the age of three months, the patient had been experiencing frequent post-feeding emesis, sometimes projectile, with variable volume and timing. Multiple formula changes have been attempted due to ongoing symptoms. He was receiving 4 oz (approximately 118.3 mL) of AR Enfamil every two hours but continued to exhibit poor weight gain. His birth weight was 3.47 kg, and at admission, he weighed 6.62 kg (5.4th percentile). On examination, he exhibited truncal weakness and bilateral symmetric lower extremity weakness. Upper extremity strength was intact (5/5), and he was able to push up during tummy time with appropriate head control. He tracked objects up to 180 degrees.

Due to concerns for failure to thrive, a comprehensive work-up was initiated (Table 1). Laboratory findings included a normal white blood cell (WBC) count with no left shift, hemoglobin of 12.3 g/dL, and platelets of 503 × 10^9^/L. A complete metabolic panel showed low bicarbonate 20.0 mEq/L and chloride 108 mEq/L due to dehydration, which normalized after rehydration. Liver enzyme levels remained elevated: aspartate aminotransferase (AST) of 219 U/L and alanine transaminase (ALT) of 227 U/L, with normal alkaline phosphatase and bilirubin. Amylase, lipase, magnesium, and albumin were within normal limits. The hepatitis panel, viral panel, and thyroid profile were negative. An abdominal ultrasound was performed due to suspected pyloric stenosis, but there was no evidence of hypertrophic pyloric stenosis or bile duct dilation. Gastric contents were seen passing into the duodenum, ruling out a gastric outlet obstruction. Due to a negative GI workup, further evaluation for lysosomal storage diseases, glycogen storage diseases, and muscular dystrophies was conducted (Table 2).

**Table 1:** Initial laboratory values on admission ALT: alanine transaminase; ALP: alkaline phosphatase; AST: aspartate aminotransferase

Test	Result	Reference Range	Comments
White Cell Count	9.98 × 10⁹/L	5–19 × 10⁹/L	Normal, no left shift
Hemoglobin	12.3 g/dL	9.5–14 g/dL	Normal
Platelets	503 × 10⁹/L	150–450 × 10⁹/L	Mildly elevated
Bicarbonate	20.0 mEq/L	16–24 mEq/L	Low-Normal
Chloride	108 mEq/L	96–110 mEq/L	High-Normal
AST	170 U/L	15–60 U/L	Elevated
ALT	180 U/L	10–50 U/L	Elevated
ALP	217 U/L	80–600 U/L	Normal
Bilirubin	0.3 mg/dL	0.1–1.2 mg/dL	Normal

**Table 2:** Repeat and specialized laboratory values ALT: alanine transaminase; AST: aspartate aminotransferase

Test	Result	Reference Range	Comments
AST (Repeat)	219 U/L	15–60 U/L	Persistently elevated
ALT (Repeat)	227 U/L	10–50 U/L	Persistently elevated
Creatine Kinase (CK)	6988 U/L	<200 U/L	Markedly elevated
Aldolase	>56 U/L	<7.6 U/L	Markedly elevated
Alanine (Plasma)	172.4 µmol/L	200–500 µmol/L (approx)	Low

**Figure 1:**
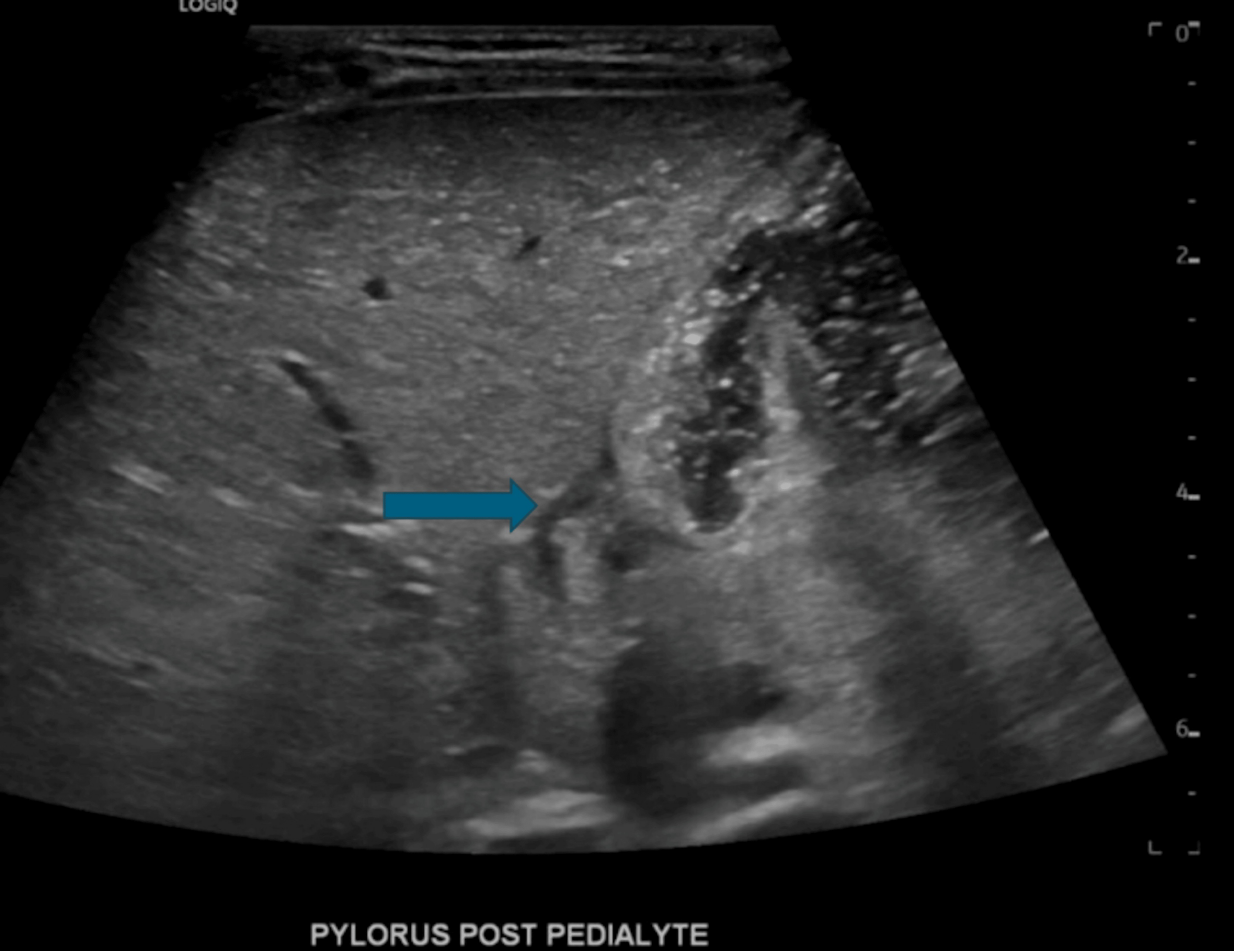
Gastric content passing into the duodenum

Given the significantly elevated creatine kinase (CK: 6,988 U/L) and aldolase (>56 U/L), along with a low alanine level (172.4 µmol/L), genetic testing was pursued to evaluate for an underlying neuromuscular disorder. Pediatric gastroenterology and neurology were consulted due to the suspicion of DMD. MRI brain and echocardiography were performed to evaluate any central nervous system (CNS) abnormalities associated with developmental delay and early cardiac manifestations of DMD. All the results were unremarkable. Genetic testing confirmed a hemizygous DMD mutation. Figure 1 shows the gastric content passing into the duodenum.

Following adjustments to his feeding regimen, the patient tolerated 4 oz of AR Enfamil with minimal spit-up and was able to consume small amounts of solid baby food, although only a few bites. He was discharged on 5 oz of AR Enfamil with a fortifier to increase caloric intake as per nutritional recommendations. A long-term follow-up with neurology and the Muscular Dystrophy Association (MDA) clinic was arranged for continued care. At discharge, his weight was 6.69 kg, and his total hospital stay lasted seven days. This early genetic confirmation of DMD following unexplained GI symptoms highlights the need for broader differential considerations in infants with persistent transaminitis.

## Discussion

ALT and AST are enzymes commonly associated with hepatocellular injury, but they are also found in the membranes of the skeletal muscles, heart, liver, and intestines [5]. This broader tissue distribution explains the elevation of these enzymes in muscular dystrophies such as DMD. Their elevation in infants, especially when unaccompanied by other signs of liver disease, presents a valuable opportunity to consider neuromuscular conditions before more overt signs such as delayed milestones or motor weakness emerge [1,6].

DMD is caused by mutations in the dystrophin gene, a large and mutation-prone gene that encodes the dystrophin protein essential for muscle membrane stability and repair. In the absence of functional dystrophin, repeated muscle contraction leads to membrane damage, fibrosis, and replacement with fatty tissue, manifesting physically as signs such as Gower’s sign, where children use their hands to push off their thighs when rising from the floor [7,8]. The disease is often asymptomatic in infancy, with caregivers only noticing subtle delays in motor development or ambulation.

While CK remains the most sensitive and specific biomarker for muscular dystrophies, elevations in AST and ALT can serve as early, indirect indicators [9]. Muscle membrane damage increases sarcolemmal permeability, leading to leakage of CK, AST, ALT, and other intracellular proteins into the plasma [2,8]. Isolated elevation of AST and ALT is often misattributed to liver pathology, but the absence of elevated bilirubin, alkaline phosphatase, or gamma-glutamyl transferase (GGT) should prompt further neuromuscular evaluation, including CK screening, as emphasized in recent studies [1,3].

For definitive diagnosis, additional investigations, such as muscle biopsy, electromyography (EMG), and genetic analysis, are necessary. Early identification is crucial, as prompt initiation of corticosteroid therapy has become the cornerstone of treatment, delaying disease progression and preserving muscle function in the late teens. Moreover, physical therapy and structured exercise programs play a supportive role in maintaining functional capacity and enhancing patient outcomes [10].

This case emphasizes broadening the differential diagnosis, especially in infants with unexplained transaminitis; recognizing atypical presentations of DMD; initiation of therapy; and improved prognosis.

## Conclusions

This case underscores the need to recognize atypical DMD presentations, particularly in infants with GI symptoms and unexplained transaminitis. It highlights the importance of CK testing in cases of isolated AST/ALT elevation with a negative GI workup. DMD should be considered in failure-to-thrive evaluations with delayed motor milestones, especially with a relevant family history. Early genetic diagnosis is crucial for timely intervention and improved long-term outcomes.
